# Role of Demyelination Efficiency within Acellular Nerve Scaffolds during Nerve Regeneration across Peripheral Defects

**DOI:** 10.1155/2017/4606387

**Published:** 2017-03-21

**Authors:** Meiqin Cai, Tengchao Huang, Bo Hou, Ying Guo

**Affiliations:** Departments of Neurosurgery, The Third Affiliated Hospital, Sun Yat-sen University, Guangzhou, Guangdong 510630, China

## Abstract

Hudson's optimized chemical processing method is the most commonly used chemical method to prepare acellular nerve scaffolds for the reconstruction of large peripheral nerve defects. However, residual myelin attached to the basal laminar tube has been observed in acellular nerve scaffolds prepared using Hudson's method. Here, we describe a novel method of producing acellular nerve scaffolds that eliminates residual myelin more effectively than Hudson's method through the use of various detergent combinations of sulfobetaine-10, sulfobetaine-16, Triton X-200, sodium deoxycholate, and peracetic acid. In addition, the efficacy of this new scaffold in repairing a 1.5 cm defect in the sciatic nerve of rats was examined. The modified method produced a higher degree of demyelination than Hudson's method, resulting in a minor host immune response in vivo and providing an improved environment for nerve regeneration and, consequently, better functional recovery. A morphological study showed that the number of regenerated axons in the modified group and Hudson group did not differ. However, the autograft and modified groups were more similar in myelin sheath regeneration than the autograft and Hudson groups. These results suggest that the modified method for producing a demyelinated acellular scaffold may aid functional recovery in general after nerve defects.

## 1. Introduction

The current gold standard of treatment for peripheral nerve defects remains autologous nerve transplantation [1], but the application of this approach in clinical practice is hindered by limited sources and poor individual matching [2–4]. Moreover, the use of allogeneic nerve grafts is primarily limited by immune rejection, but pretreating the allograft nerve with chemical extraction can reduce its immunogenicity [5–7]. Furthermore, allogeneic nerve grafts feature complex ultrastructures, provide a contact site of cell recognition, and maintain the extracellular matrix required for nerve tissue regeneration, which is an ideal substitute material for nerves [8]. Specifically, Sondell et al. reported a simple method for the preparation of decellularized nerve scaffolds using sciatic nerve tissue treated with Triton X-100 and sodium deoxycholate [9]. This method removed nerve cells and myelin to reduce the immune response to the nerve xenografts. However, this method did not completely preserve the active components of the graft, such as the basal laminar tube in the nerve tissue and other structures, resulting in a poor repair efficacy for the treatment of nerve injuries [10–13]. In 2004, Hudson et al. proposed a modified method and showed it to be superior to the Sondell method, as evidenced by the complete preservation of the basement membrane and structural components and improved nerve regeneration after the transplantation [5, 9]. Thus, Hudson's method has gradually become the most commonly used method for the preparation of acellular peripheral nerve scaffolds.

However, electron microscopy images of Hudson's acellular nerve scaffolds (hANSs) showed residual myelin in the nerve tissue that may impede the extension of the regenerative axons and affect nerve repair.

To solve the above problems, we modified Hudson's method to remove myelin components more thoroughly and prepare demyelinated acellular nerve scaffolds (dANSs) with a high degree of demyelination. Furthermore, the ability of these 2 acellular scaffolds to repair nerves was investigated.

## 2. Materials and Methods

### 2.1. Animals

All experimental animals were housed under standard conditions, and all protocols were conducted in accordance with the recommendations of the Experimental Animal Center of Sun Yat-sen University. Eighteen adult male 10-week-old Sprague-Dawley rats weighing between 300 g and 350 g were used. The animals were housed in a temperature-controlled environment at 21 ± 1°C under a 12 h light/12 h dark cycle with free access to food and water.

Table 1 provides an overview of the groups, which consisted of 6 animals each.

### 2.2. Preparation of Acellular Nerve Grafts

#### 2.2.1. Preparation of Demyelinated Acellular Nerve Scaffolds

The raw materials of the scaffolds were harvested from the sciatic nerves of SD rats that were sacrificed for other purposes. The adipose tissue and the collagenous connective tissue surrounding the nerve were removed, and the nerve tissue was cut into 1.5 cm-long segments and washed in distilled water. The nerves were then placed in deionized distilled water (ddH_2_O) for 6 h, followed by incubation in 125 mM sulfobetaine-10 (SB-10) in ddH_2_O with agitation for 12 h. The nerve segments were washed in phosphate-buffered saline (PBS) (10 min ×3) and agitated in 0.14% Triton X-200 and 0.6 mM sulfobetaine-16 (SB-16) in ddH_2_O for further decellularization for 24 h. The samples were rinsed 3 times in PBS followed by one 10 min wash. The tissue was then immersed in 125 mM SB-10 in ddH_2_O with agitation for 12 h. The extract medium was then changed to 4% sodium deoxycholate, and the tissue was agitated for 24 h. Finally, the nerve segments were rinsed in PBS (2 h ×3). For sterilization [14], nerves were immersed in 0.1% (v/v) peracetic acid (PAA) (Adamas-Beta, Switzerland) diluted in ddH_2_O for 3 h followed by 3 washes in sterile PBS for 1 h. All washing steps were performed at room temperature.

All chemicals were purchased from Sigma (St. Louis, MO) unless otherwise noted.

#### 2.2.2. Preparation of hANSs

hANSs were prepared based on the chemical decellularization protocol published by Hudson et al. [5]. In brief, the nerve tissue was treated with a series of detergents, including SB-10, SB-16, and Triton X-200, and ddH2O. All steps were performed in a laminar flow hood to maintain sterility. The hANSs were then stored in PBS until use.

### 2.3. Characterization of ANSs

#### 2.3.1. Characterization of Residual DNA in the Sciatic Nerve Scaffolds

Qualitative and quantitative assessments of residual DNA were evaluated against established criteria [15, 16]: (1) the absence of visible nuclei in hematoxylin and eosin (H&E) or 4′,6-diamidino-2-phenylindole- (DAPI-) stained sections and (2) no DNA fragments exceeding 200 bp in length.

*(1) Qualitative Assessment.* Acellular scaffolds were embedded in Tissue-Tek Optimum Cutting Temperature (OCT) Compound (Sakura Finetek, USA), sectioned at a thickness of 10 *μ*m, and captured on poly-L-lysine-coated slides. The tissues were then stained with H&E, and images were visualized using a light microscope (Nikon Eclipse-80, Japan). 

*(2) Quantitative Assessment.* Acellular grafts were digested with 0.1 mg/mL proteinase K for 1 h at 55°C. The samples were then repeatedly extracted with phenol/chloroform and centrifuged to remove protein until the interface was free of white material. The aqueous phase extract was then mixed with 3 M sodium acetate and 100% ethanol and centrifuged to pellet DNA. The pellet DNA was rinsed with 70% ethanol, centrifuged, and dried. A 1.0% agarose gel containing ethidium bromide was then loaded with extracted DNA and electrophoresed for 2 h at 80 V, and the DNA was then visualized with ultraviolet transillumination.

#### 2.3.2. Characterization of Residual Myelin and the Basal Lamina Tube

The acellular scaffolds were stained with toluidine blue staining and examined under a transmission electron microscope (TEM). Individual samples were fixed in 2.5% (m/v) glutaraldehyde in 0.1 M PBS (pH 7.2) for 2 h and postfixed for 90 min in 1% osmium tetroxide. The sections were dehydrated in an ascending series of ethanol solutions, stained with 1% uranyl acetate overnight, embedded in a Poly/Bed 812 resin (Ted Pella, CA, USA), and polymerized at 60°C for 48 h. Cross-sections (1 *μ*m thick) were cut and stained with toluidine blue. Ultra-thin cross-sections (60–70 nm) were obtained and stained in lead citrate and uranyl acetate. The ultrastructure of the scaffold was subsequently observed using a TEM (FEI TECNAI SPIRIT G 2).

Acellular scaffolds were embedded in Tissue-Tek® OCT Compound (Sakura Finetek, USA), sectioned at a thickness of 10 *μ*m, and captured on poly-L-lysine-coated slides. After citrate antigen retrieval and blocking with 2% normal goat serum (Dako, Denmark), the sections were immunostained for laminin by incubation with rabbit anti-rat laminin primary antibody (1 : 1000, Sigma-Aldrich, USA), followed by incubation with goat anti-rabbit IgG Alex flour 594-conjugated secondary antibody (1 : 800, Jackson Immuno-research). The stained sections were then imaged under a fluorescence microscope (Eclipse-80i, Olympus, Japan).

### 2.4. Experimental Design and Surgical Procedure

The animals in each group underwent identical surgical procedures: under general anesthesia with pentobarbital (60 mg/kg, given intraperitoneally) and aseptic conditions, an incision was made in the skin from the left knee to the hip to expose the underlying muscles, which were then retracted to reveal the sciatic nerve [3]. A 1.5 cm nerve segment was completely transected with fine surgical scissors at 0.3–0.5 cm in front of the tibial peroneal nerve bifurcation of the sciatic nerve as a starting point, extending 1.5 cm toward the proximal end. The freshly trimmed 1.5 cm acellular grafts were placed and sutured to the proximal and distal nerve stumps using a sleeve technique. In the control group, a 1.5 cm segment was completely excised, reversed, and resutured on each side. All epineural sutures consisted of 10/0 monofilament nylon and were placed using an operating microscope (Carl-Zeiss, Germany).

### 2.5. Immunological Analysis

One day prior to the surgery as well as 1 and 8 weeks after surgery, 0.2 mL of peripheral venous blood was drawn from the tail of each rat into a lithium-heparin tube. Lysis buffer (2 mL, BD Bioscience, USA) was then added to remove the red blood cells. Peripheral blood mononuclear cells (PBMCs) were isolated by density centrifugation for 5 min at 800 rpm using Lymphoprep (Nycomed Pharma AS, Oslo). The PBMCs were then incubated for 10 min at room temperature with combinations of the following monoclonal antibodies: CD3-FITC, CD4-PerCP-CY5.5, and CD8-PE (BD Bioscience, USA). The PBMCs were then washed twice in PBS, bovine serum albumin, and azide before being fixed in 1% paraformaldehyde. The cells were viewed on a BD FACSCalibur Flow Cytometer, and the raw data were analyzed using BD Cell Quest software.

### 2.6. Evaluation of Nerve Regeneration

#### 2.6.1. Thermosensitivity

The thermosensitivity was assessed starting at the beginning of the 2nd postoperative week based on the ability of rats to actively spread their digits and their sensitivity to 55°C hot water (the test was positive if the rats retracted their legs from the water within 3 s). Two blinded observers recorded the movements every week throughout the 8-week period. Any disagreement was resolved unanimously by discussion.

#### 2.6.2. Gastrocnemius Muscle Weight Ratio

The weight ratio was calculated and served as an indirect measurement of muscle regeneration after nerve repair. Rats in all groups were sacrificed 8 weeks after surgery. Using an operating microscope, both the left and right (control) gastrocnemius muscles were carefully cleaned and dissected, dividing their tendinous origin and insertion from the bone. The muscles were weighed following harvest, and the gastrocnemius muscle weight ratio (experimental/control, *E*/*C*) was calculated for each rat based on the weight of the gastrocnemius muscle from the experimental leg (left leg) versus the normal leg (right leg) according to the following formula:

(1) $$\begin{array}{c} \frac{E}{C}=\frac{weight\;\;experimental}{weight\;\;control\;\;muscle}. \end{array}$$

#### 2.6.3. Morphological Evaluation of Regenerated Nerve Segments

Repaired left sciatic nerve specimens were harvested during week 8, and 0.5 cm midpoint sections of 3 grafts were stained with toluidine blue and Neurofilament-200 (NF-200). The toluidine blue staining step was described previously. The NF-200 staining step was performed as follows. The acellular scaffolds were embedded in Tissue-Tek® OCT Compound (Sakura Finetek, USA), sectioned at a thickness of 10 *μ*m, and captured on poly-L-lysine-coated slides. After blocking with 2% normal goat serum (Dako, Denmark), the sections were incubated with rabbit anti-rat NF-200 (1 : 2000, Sigma-Aldrich, USA) at 4°C overnight; two-step visualization was performed using GTvision immunohistochemical detection kit (Gene Tech; Shanghai) with hematoxylin counterstaining. The structures of the regenerated nerves were subsequently observed using by light microscopy.

The number of nerve fibers (*n*), myelin thickness (MT), and diameter of myelinated fibers (FD) were observed using toluidine blue staining and TEM mentioned above, and *g*-ratio (axon to fiber diameter) was obtained from 5 representative fields in TEM images to quantitatively assess the specimens using Image ProPlus Imaging software 4.5 (Media Cybernetics, Marlow, UK). The *g*-ratio was calculated according to the following formula [3]:

(2) $$\begin{array}{c} g\text{-}ratio=\frac{\left(FD-2*MT\right)}{FD}. \end{array}$$

### 2.7. Statistical Analysis

All data are expressed as the mean ± standard deviation (SD) for independent samples from the 3 groups. The data were analyzed using the SPSS 16.0 software (SPSS Inc., Chicago, IL, USA). One-way analysis of variance (ANOVA) and Bonferroni's test were used to statistically compare the various groups, and statistical significance was set at *p* < 0.05.

## 3. Results

### 3.1. Efficiency of Decellularization

**Figure 1:**
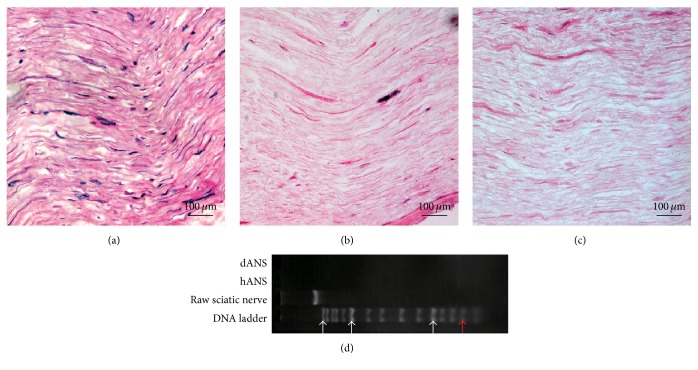
Characterization of residual DNA in the scaffolds. After H&E staining, cell nuclei were visible in (a) native sciatic nerve tissue but not in (b) hANSs or (c) dANSs. (d) Gel electrophoresis showed that the residual DNA fragments did not exceed 200 bp in hANSs or dANSs. White arrows denote (left-right) 10000 bp, 3000 bp, and 500 bp. The red arrow denotes 200 bp.

No residual nuclei were visible in the H&E images of samples prepared using the 2 methods (Figures 1(a), 1(b), and 1(c)). Moreover, the maximum fragment size of residual DNA from both acellular scaffolds did not exceed 200 bp (Figure 1(d)).

### 3.2. Residual of Myelin and Laminin

**Figure 2:**
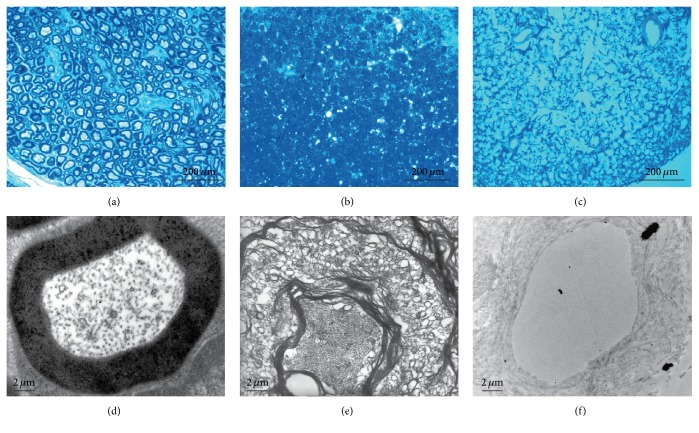
Characterization of residual myelin. Myelin was present in (a) raw sciatic nerve tissue, (b) hANSs, and (c) dANSs by toluidine blue staining. TEM showed that the myelin sheath was looser in (e) hANSs than (d) raw sciatic nerve tissue but remained attached to the basal lamina tube. TEM of (f) dANSs revealed no apparent myelin.

The residual myelin was evaluated using toluidine blue and TEM (Figures 2(a), 2(b), and 2(c)). Specifically, the peripheral nerve was free of myelin in the dANSs, whereas hANSs showed myelin that had been slightly loosened from the raw nerve. TEM images of hANSs and dANSs (Figures 2(d), 2(e), and 2(f)) showed that the dANSs were free of myelin and consisted of an empty basal laminal tube, whereas myelin fragments remained in the basal laminal tube in the hANSs.

**Figure 3:**
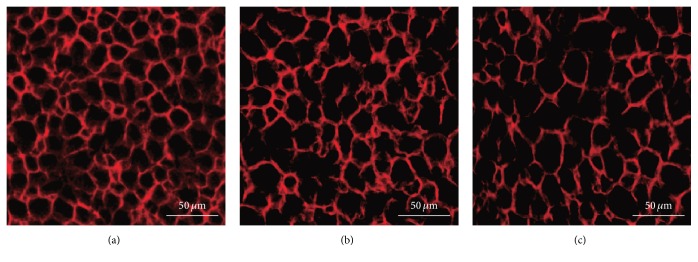
Analysis of the basal lamina tube. Laminin staining of the (a) raw sciatic nerve and scaffolds revealed that the integrity of the basal lamina tube was well preserved both in (b) hANSs and in (c) dANSs.

The preserved basal laminal tube was identified by immunostaining samples after decellularization. Both hANSs (Figure 3(b)) and dANSs (Figure 3(c)) consisted primarily of laminin, the main component of the basal lamina tube, which remained intact. Moreover, laminin was arranged in an ordered manner, which indicated that the 3D structure of the natural nerve remained mostly intact after the 2 decellularization processes.

### 3.3. General Observation of Animals

All experimental animals survived the surgery, and the wounds generally healed without symptoms of inflammation or discomfort. The left limbs exhibited dysfunction after surgery and regained different levels of function 8 weeks after surgery. The ANSs were partially degraded and had integrated well into the host tissue. Neither scaffold dislocation nor neuroma formation was evident after 8 weeks.

### 3.4. CD3+/CD4+/CD8+ Peripheral Blood Flow Cytometric Analysis

To test the immunological response of the host to ANSs in vivo, we harvested CD3+ mononuclear cells from the peripheral blood at 3 time points. CD4+/CD8+ was selected to evaluate the immunological response of the host.  Table 2 shows no significant differences in markers of inflammation between the dANSs group and the control group (*p* > 0.05), whereas the proportion of CD4+/CD8+ cells was slightly higher in the hANS group than in the control group and dANS group 1 week after surgery (*p* < 0.05); however, this difference decreased by the 8th week.  Table 3 shows the total numbers of CD4+ and CD8+ T lymphocytes among the 3 groups. The pattern of change of the total number of T lymphocytes was similar to that of the ratio of CD4+/CD8+ T lymphocytes. The total number of T lymphocytes was significantly higher in the hANS group than in the control group and dANS group (*p* < 0.05); however, no differences among the 3 groups were observed by the 8th week.

### 3.5. Functional Outcome

#### 3.5.1. Thermosensitivity

Table 4 shows that thermosensitivity was restored at different time points within the ANS groups, that is, after 4 weeks in the dANS group and after 5 weeks in the hANS group. The control group showed a positive reaction at the beginning of the 2nd week.

#### 3.5.2. Muscle Weight Ratio

**Figure 4:**
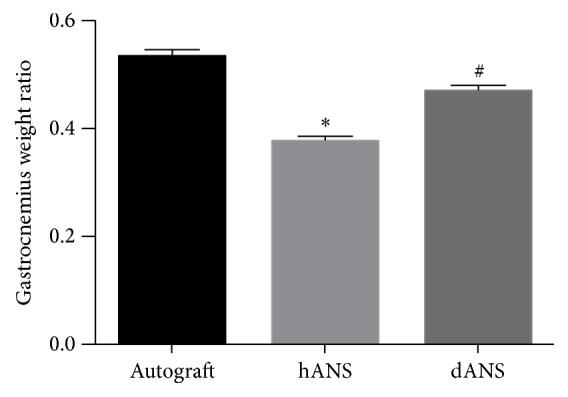
Graph showing the mean gastrocnemius muscle weight ratio (experimental/control, *E*/*C*) of the 3 groups. Values are expressed as the mean ± SD, ^*∗*^Compared with autograft group, *p* < 0.05; ^#^compared with dANS group, *p* < 0.05.

As shown in Figure 4, the gastrocnemius muscle weight ratios were 0.54 ± 0.06, 0.38 ± 0.08, and 0.47 ± 0.06 in the autograft group, hANS group, and dANS group. The muscle weight ratios in the autograft group and dANS group were both significantly higher than that in the hANS group (*p* < 0.05), and there were no significant differences between the ratios of the autograft group and dANS group (*p* > 0.05).

### 3.6. Morphological Evaluation of Nerve Regeneration

**Figure 5:**
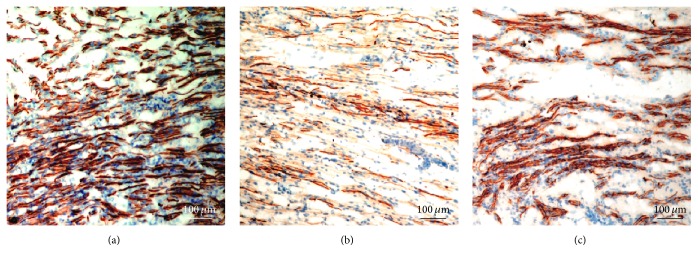
The results of NF-200 staining showed the regenerated neurofilament in all three groups of grafts. (a) The nerve axons of the autograft group were thick in a dense arrangement. (b) The regenerated nerve axons of the hANS group were sparse in arrangement and small in morphology. (c) The morphology and arrangement of the regenerated nerve axons of the dANS group were closer to (a) than the hANS group in (b).

After repairing the 1.5 cm sciatic nerve defect, a histological examination was performed to detect nerve regeneration. NF-200 staining revealed differences in the penetration of regenerated axons within each graft (Figures 5(a), 5(b), and 5(c)). The regenerated axons were thicker and denser in the autograft group (Figure 5(a)) and dANS group (Figure 5(c)) than in the hANS group (Figure 5(b)).

**Figure 6:**
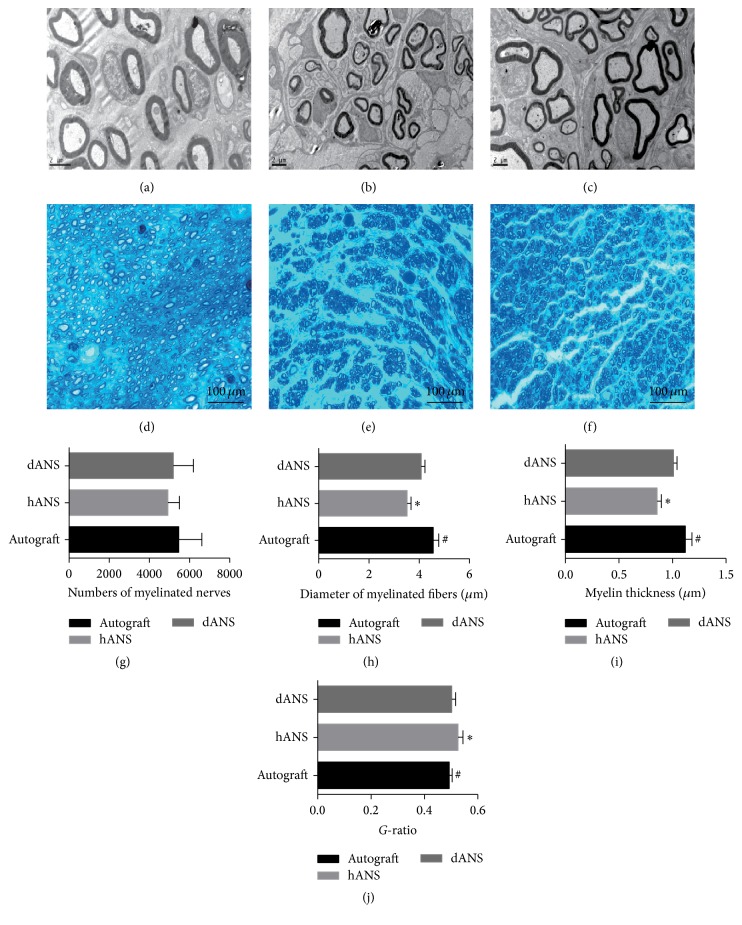
Morphological evaluation of the regenerated nerve. TEM showed that the myelin sheath was larger and thicker in (a) the control group and (c) dANS group than (b) the hANS group. Myelin was present in (a) the control group, (b) hANS group, and (c) dANS group by toluidine blue staining. The hANS group had more irregular myelinated fibers in minicompartments. For myelin analysis, (g) the number of myelinated fibers, (h) the diameter of myelinated fibers, (i) the myelination thickness, and (j) the *g*-ratio were quantitatively evaluated and compared by statistical analysis. Values are expressed as the mean ± SD. ^*∗*^Compared with the autograft group, *p* < 0.05. ^#^Compared with dANS group, *p* < 0.05.

Moreover, the samples were stained with toluidine blue and examined under a light microscope and a TEM; we evaluated the regeneration of myelinated nerves by examining semithin and ultra-thin cross-sections. Pictures from midpoint sections of grafts reveal a clear difference among all groups (Figures 6(a), 6(b), 6(c), 6(d), 6(e), and 6(f)).

Cross-sections from the hANS group showed nerve regeneration, as evidenced by a larger number of thin and irregular myelinated fibers in minicompartments than in the dANS and control groups. The average diameters of myelinated fibers (Figure 6(h)) were significantly larger in the dANS and control groups compared with the hANS group, and the values between the dANS group and control group did not significantly differ. The average myelination thickness (Figure 6(i)) was significantly larger in the dANS and control groups than in the hANS group, whereas the values did not significantly differ between the dANS and control groups. The result for the *g*-ratios (Figure 6(j)) was consistent with the results above. However, the number of myelinated fibers (Figure 6(g)) did not significantly differ between the 3 groups (*p* < 0.05).

## 4. Discussion

In tissue transplantation, immunological rejection is associated with the presence of the major histocompatibility complex (MHC) in tissues [17]. In peripheral nerve tissue, the components containing the MHC mainly include Schwann cells, the myelin sheath, and axons, and the antigenicity of other structures, such as the basal laminar tube, is very weak [18].

The Hudson method has been shown to achieve good results in removing the cellular components and preserving the integrity of the basal laminar structure. The demyelination effect was evaluated using Western blotting for myelin basic protein (MBP) and using lipid fluorescence staining [5]. However, MBP comprises only 1/3 of the myelin proteins in peripheral nerve tissue and cannot reflect the total myelin protein content [19]. Moreover, the TEM observations in this study showed that most basal laminar tubes included residual disintegrating myelin debris. Therefore, the conditions of decellularization do not effectively remove the myelin sheath, and the extraction conditions require improvement [20, 21].

The choice of chemical extraction medium directly affects the extraction efficacy. The following agents are commonly used as extraction media: (1) nonionic compounds, such as Triton X-100; (2) amphoteric compounds, such as SB-10 and SB-16; and (3) anionic compounds, such as Triton X-200, sodium dodecyl sulfate (SDS), and PAA [14]. Among these compounds, nonionic and amphoteric agents preserve the structure of the neural tube well, whereas anionic compounds remove cellular components and structures well [22]. Because the surface activity of existing cationic compounds is too strong, they easily damage structural proteins, such as laminin, and are strongly cytotoxic. Therefore, they were not tested. The classical Hudson method relies on 2 amphoteric compounds, SB-10 and SB-16, and an anionic compound, Triton X-200, whose extraction efficacy is moderate. These compounds do not effectively remove the myelin lamellar structure attached to the basal laminar tube. Therefore, in addition to extracting SB-10, SB-16, and Triton X-200, the anionic extraction compound sodium deoxycholate, which has shown high extraction efficacy, was added to further destroy cellular components. Moreover, PAA is commonly used to extract and disinfect biological implant materials. PAA can dissolve the cytoplasm and nucleic acids while simultaneously disinfecting the material due to its oxidation effect. Hodde and Voytik-Harbin studied the effect of various types of disinfectants on the extracellular matrix scaffold and found that, in addition to sterilizing the scaffold, PAA can also protect the active components (basic fibroblast growth factor (b-FGF) and vascular endothelial growth factor (VEGF)) of the scaffold and maintain the internal complex 3D structure of the scaffold [14, 23]. At the end of the experiment, PAA was used to further remove the cell debris and the loose myelin components while simultaneously disinfecting the scaffold.

In this experiment, the results of DNA electrophoresis, H&E, and immunofluorescence staining indicated that the degree of decellularization and the integrity of the basement membrane were comparable for the dANSs prepared by us and the hANSs [16]. The TEM images showed that myelin was more thoroughly removed from the dANSs; specifically, the lamellar tight arrangement of the myelin sheath on the basal laminar tube was absent, and only the basal laminar tube remained. Conversely, the hANS TEM images showed that the myelin sheath was looser and thinner than before, but a small amount residual myelin remained attached to the basal laminar tube. These results suggested that the extraction method proposed herein more thoroughly removed the cellular components, especially the myelin sheath structure.

Subsequently, the effects of the dANSs and hANSs on neural repair were compared based on 3 aspects: immunological rejection, neurological functional recovery, and morphology after transplantation.

CD3+ T lymphocytes are primarily responsible for transplant rejection, among which CD4+ cells are helper/induced T lymphocytes and CD8+ cells are cytotoxic/killing T lymphocytes. Moreover, the total numbers of CD4+ and CD8+ T lymphocytes and the ratio of CD4+/CD8+ T lymphocytes are important indicators of the immune status of the body [17, 24]. In this experiment, the total number of T lymphocytes and the CD4+/CD8+ ratio was significantly lower in the autograft group and dANS group during the early stage (1st week) than in the hANS group. As healing progressed (after 8 weeks), the indicators of rejection did not differ between groups. We speculated that the immunogenicity of the tissue was decreased due to the high degree of demyelination in the dANS, and the implant consequently resulted in minimal immune rejection [25]. Due to the residual myelin components in the hANSs, the local macrophages invaded the basal laminar tube to clear the MHC during the early stage of healing. Because of the small amount of implants, the residual myelin and other antigenic substances left behind by the Hudson method were cleared during the late stage of healing, and systemic immune rejection was decreased. This result shows that the dANS is not rejected and does not produce a significant inflammatory response compared with the hANSs.

Neurological effects were assessed based on thermosensitivity and the gastrocnemius muscle weight ratio. Specifically, in rats whose sciatic nerve was repaired with a dANS, thermosensitivity returned within an average of 4 weeks, whereas this recovery period was 5 weeks in the hANS group. Moreover, the muscle ratio of the dANS group was better than that of the hANS group. We speculate that the proximal nerve fibers can more easily reach the distal end of the graft when repairing sciatic nerve defects with a dANS than with a hANS, thus rebuilding an effective nerve connection and restoring the function of the nerve [26, 27].

Cross-sections of the nerves from the 3 groups were examined. We attributed the difference in NF-200 staining between the hANSs and dANSs to a decrease in myelin residue, which may have led to a decline in the local inflammatory reaction and an improved local microenvironment, ultimately enhancing the regeneration of axons in the dANS group, which showed regeneration along the clean basement membrane. Moreover, toluidine blue staining and TEM showed that the average diameter of the regenerated myelinated nerve fibers was larger in the dANS group than the hANS group and featured a thicker myelin sheath, whereas the number of regenerated axons did not significantly differ between groups. These results further confirmed our speculations based on the observed functional recovery. A dANS is more suitable for the repair of nerve defects than a hANS because, although the number of regenerated axons did not differ between groups, the dANS is more conducive to the growth of the regenerated axons in the basal laminar tube and favors the regeneration of Schwann cell-wrapped axons to generate more nerve fibers with effective signal conduction.

Based on this analysis and previous work, functional recovery varies after microsurgical nerve repair, and sensory and motor dysfunctions are the most likely defects and result from a loss of innervation of functional neurons. Initial growth into an ANS surely contributes to sparse reinnervation [28]. However, in the early stage of the surgical repair, local immune reactions against antigens on the acellular scaffold hinder reinnervation and destroy the basal lamina [20, 29]. We attributed this immune reaction to components within the scaffold and consequently modified Hudson's method to eliminate most of the residual myelin, which was confirmed to constitute the MHC in nerve tissue. The low immunogenicity of the acellular scaffold considerably improved effective axonal penetration into the center of the scaffold, and even marginal increases in nerve regeneration can result in significant improvements in clinical function.

However, we focused solely on minor immune reactions and improvements in nerve regeneration, and mechanisms underlying these effects remain to be elucidated. Nevertheless, the decrease in the host immune response and increase in myelinated axons of the regenerating nerve are important features of the dANS, irrespective of underlying mechanisms. Further investigations should be carried out to identify different growth promoting substances and develop an ideal graft for regenerating nerves.

## 5. Conclusion

In conclusion, the current study developed an effective ANS that included less myelin than conventional scaffolds and produced minor host immune responses in vivo to provide an improved environment for nerve regeneration. Furthermore, this new method, which used different detergents to produce dANSs derived from SD rats, was evaluated using a SD rat sciatic nerve defect model. These results preliminarily support the potential use of dANSs for nerve regeneration.

Additionally, long-term and thorough neurological studies are required to assess the full potential of this dANS preparation for functional recovery.

## Figures and Tables

**Table 1:** 

Group	Description
I	dANS
II	hANS
III	Nerve autograft (control)

**Table 2:** Ratio of CD4+/CD8+ T lymphocytes at different time points (*n* = 6, mean ± SD).

Group	Preoperative	1 week postoperatively	8 weeks postoperatively
Autograft	1.632 ± 0.723	1.698 ± 0.446	1.645 ± 0.316
hANS	1.595 ± 0.658	1.962 ± 0.712^*∗*,#^	1.621 ± 0.534
dANS	1.628 ± 0.616	1.783 ± 0.548	1.693 ± 0.433

^*∗*^Compared with autograft group, *p* < 0.05. ^#^Compared with dANS group, *p* < 0.05.

**Table 3:** Total number of T lymphocytes at different time points (*n* = 6, mean ± SD).

Group	Preoperative	1 week postoperatively	8 weeks postoperatively
Autograft	2.344 ± 0.921	2.630 ± 0.974	2.577 ± 0.798
hANS	2.493 ± 1.002	2.948 ± 1.414^*∗*,#^	2.760 ± 0.862
dANS	2.459 ± 0.943	2.667 ± 1.265	2.619 ± 0.856

^*∗*^Compared with autograft group, *p* < 0.05. ^#^Compared with dANS group, *p* < 0.05.

**Table 4:** Timetable of the return of thermal sensitiveness.

Weeks	Autografts						hANS						dANS					
	1	2	3	4	5	6	1	2	3	4	5	6	1	2	3	4	5	6
2	+	−	+	−	−	+	−	−	−	−	−	−	−	−	−	−	−	−
3	+	−	+	+	−	+	−	−	−	−	−	−	−	−	−	−	−	−
4	+	+	+	+	+	+	−	−	−	−	−	−	+	+	−	+	−	−
5	+	+	+	+	+	+	+	+	−	−	+	−	+	+	−	+	−	+
6	+	+	+	+	+	+	+	+	+	−	+	+	+	+	+	+	+	+
7	+	+	+	+	+	+	+	+	+	+	+	+	+	+	+	+	+	+
8	+	+	+	+	+	+	+	+	+	+	+	+	+	+	+	+	+	+

(−) no sensitiveness; (+) positive reaction.
